# Pressure to breastfeed among mothers of infants in Poland. A cross-sectional study

**DOI:** 10.3389/fpubh.2026.1680978

**Published:** 2026-03-16

**Authors:** Piotr Kordel, Bartosz Burchardt, Klaudia Dolińska-Kaczmarek, Katarzyna Dondajewska, Maciej Kokociński, Szymon Rzepczyk, Paweł Świderski, Czesław Żaba

**Affiliations:** 1 Department of Social Sciences and the Humanities, Poznan University of Medical Sciences, Poznan, Poland; 2 Department of Forensic Medicine, Poznan University of Medical Sciences, Poznan, Poland; 3 Obstetrics Ward, The Holy Family Hospital, Poznan, Poland; 4 Faculty of Sociology, Adam Mickiewicz University in Poznan, Poznan, Poland

**Keywords:** breast fed, breastfeeding, maternal health, mental health, Poland

## Abstract

**Objectives:**

This study aimed to explore the phenomenon of pressure to breastfeed among mothers of infants in Poland. Specifically, we examined the prevalence of perceived pressure, its sources, and the sociodemographic factors associated with such pressure. Additionally, we investigated whether an inability to breastfeed affects maternal self-esteem.

**Material and methods:**

A cross-sectional study was conducted with 586 mothers of infants aged 12 months or less, recruited via online parenting groups. Participants completed a 12-question survey addressing sociodemographic variables, breastfeeding encouragement, and perceived pressure to breastfeed. Statistical analyses were performed using Pearson's r, Student's *t*-test, Welch's *t*-test, and χ^2^, where applicable. The effect size was calculated using Cohen's d, Cramer's V, and Phi, where applicable.

**Results:**

Among participants, 81.2% reported breastfeeding encouragement, primarily from healthcare professionals, scientific literature, and parenting campaigns. However, 39.2% perceived this encouragement as pressure. The primary sources of pressure included personal expectations and healthcare professionals. Younger mothers, those living in rural areas, and first-time mothers were more likely to feel pressured. Notably, 57.4% of non-breastfeeding mothers experienced pressure, compared to 26.3% of breastfeeding mothers. Mothers who experienced pressure also rated themselves as lesser mothers (mean score: 7.0 vs. 7.42 among those without pressure, *p* = 0.001 on a scale from 0 to 9).

**Conclusions:**

Perceived pressure to breastfeed is prevalent in Poland, particularly among younger and first-time mothers. This pressure, coming from mothers themselves but also from healthcare professionals, can negatively impact maternal self-esteem and highlights the need for better education about infant feeding methods.

## Introduction

Breastfeeding is associated with many positive outcomes, both for the child and the mother. Better cognitive development (1–4), reduced infections (5, 6), lower asthma and obesity prevalence (3, 7), or a reduction in the risk of type 2 diabetes (3) are some of the long-term benefits for breastfed children. Successful breastfeeding can lead to post-pregnancy weight loss and protect the mother against type 2 diabetes, hypertension, and cardiovascular disease. It also lowers the risk of ovarian and breast cancer (8–11). No wonder exclusive breastfeeding is recommended as the best infant feeding method for the first 6 months of life (12), and the World Health Organization recommends continued breastfeeding through the first 2 years of life (13). Based on these recommendations, numerous global and local initiatives promoting breastfeeding have been developed and implemented (14–18), resulting in the creation of a breastfeeding culture and pressure to breastfeed in some countries (14). A study conducted in Poland indicates that as many as 97% of women start breastfeeding after childbirth. However, it also showed high rates of rapid abandonment of exclusive breastfeeding (only 43.5% of the studied mothers exclusively breastfed at 2 months after birth, 28.9% at 4 months, and 4% at 6 months), as well as the introduction of formula milk in the first day of life (15). It may stem from the fact that breastfeeding can be physically challenging and can lead to many adverse outcomes, i.e., pain while feeding, soreness after feeding, painful uterine contractions, and dry and bleeding nipples or mastitis. We must also remember that not every woman can breastfeed due to a low or no milk supply (16). Given that context, the pressure to breastfeed can have a negative impact on maternal mental health, such as anxiety, depression, or even suicide ideation (8, 17–19).

## Objectives

This cross-sectional study aimed to explore the phenomenon of pressure to breastfeed among mothers of infants (children under 12 months of age) in Poland, as, to the best of our knowledge, no research has been conducted on this topic. We aimed to investigate the frequency of perceived pressure to breastfeed among mothers of infants in Poland, identify its sources and scale, and examine which sociodemographic characteristics contribute to mothers experiencing it strongly (age, education, place of residence, and having children before). We also wanted to know whether the mothers who could not breastfeed experienced the pressure more and whether the inability to breastfeed made them rate themselves as worse mothers than those who breastfed their children.

## Material and methods

### Sample

A sample of 586 mothers of infants was collected via online social media advertising targeting parenting groups from January to November 2024. The mean age of the participants was 30.22 years (min = 18, max = 45, SD = 4.60). Forty-four percentage of participants (*n* = 258) were under 30, and 3.9% (*n* = 23) were 40 or older. For 66.6% of the study participants (*n* = 390), the infant was the first and by far the only child. The majority of participants (76.6%, *n* = 449) were university graduates, 22% (*n* = 129) were high school graduates, and only 1, 4% (*n* = 8) declared that they finished their education at the elementary level 35.8% of participants resided in rural areas, while the remaining participants lived in cities of varying sizes (see Table 1).

**Table 1:** Participants' place of residence.

**Place of residence**	***n***	***%***
Rural areas	210	35.8
Town <50,000 inhabitants	115	19.6
City 50,000–150,000 inhabitants	58	9.9
City 150,000–500,000 inhabitants	40	6.8
City >500,000 inhabitants	163	27.8
Total	586	100

### Survey design

The participants were asked to complete a survey developed by the research team. The first four questions concerned the sociodemographic variables (age, education, place of residence, and number of children). Next, we asked participants to rate themselves as mothers from 0 (a bad mother) to 9 (a great mother). Then, the women were asked whether they breastfed their children at the time of the study; if not, they were asked to specify the reason.

The remaining questions of the research survey focused on the experience of pressure to breastfeed. First, participants were asked whether they had ever been encouraged to breastfeed (yes/no). If the answer was yes, participants were asked to complete the Breastfeeding Encouragement Questionnaire, a self-report measure developed specifically for this study. It evaluates nine potential sources of encouragement to breastfeeding (see Table 2), which the participants could rate on a 10-point scale from 0 (no encouragement) to 9 (powerful encouragement). The designed measure demonstrated good internal consistency (α = 0.88), and its overall score is the mean of all nine items.

**Table 2:** Breastfeeding encouragement questionnaire items and general score (scale from 0 to 9), *N* = 476.

**Encouragement factors**	**Mean**	**SD**
Healthcare professionals	5.16	3.38
Scientific research results	4.83	3.51
Parenting literature	4.02	3.44
Breastfeeding promotion campaigns	3.56	3.37
Popular opinion	3.36	3.29
The child's father	3.00	3.32
Parents	2.80	3.12
Friends	2.15	2.72
In-laws	2.05	2.91
General score	3.44	2.32

Next, the participants were asked if they had ever experienced the feeling of pressure to breastfeed their children (yes/no). Again, if the answer was yes, the participants completed the Breastfeeding Pressure Questionnaire—another self-report measure developed by the research team, using the same 10-point scale (0—no pressure, 9—powerful pressure), and a mean general score, consisting of 10 items (see Table 3). The Breastfeeding Pressure Questionnaire presented good internal consistency (α = 0.86).

**Table 3:** Breastfeeding pressure questionnaire items and general score (scale from 0 to 9), *N* = 230.

**Pressure factors**	**Mean**	**SD**
My expectations	4.97	3.70
Healthcare professionals	4.73	3.60
Popular opinion	4.14	3.48
Breastfeeding promotion campaigns	3.05	3.44
Scientific research results	2.92	3.31
Parenting literature	2.73	3.27
In-laws	2.63	3.34
Parents	2.36	3.12
Friends	1.96	2.80
The child's father	1.91	2.92
General score	3.14	2.22

Lastly, we asked the participants if they still experienced pressure to breastfeed during the study (yes/no).

### Statistical analysis

The statistical analysis of the data was performed using IBM SPSS Statistics v.26 (IBM Corp, Amonk, NY, USA) software. The procedures included analyzing correlations between the designed measures and the mothers' self-ratings using Pearson's r. Both Questionnaires have undergone factor analysis using Direct Oblimin rotation with Kaiser normalization, preceded by the Kaiser–Meyer–Olkin (KMO) test to assess whether the data were well-suited for the procedure. The research team conducted a linear regression analysis to create a model using sociodemographic variables (age, number of children, education, and place of residence) to predict breastfeeding pressure. However, the designed model had a statistically insignificant fit (*R*^2^ = 0.02, df = 4, F140 = 1.19, *p* > 0.05); therefore, the research team decided to present the results of univariate analyses previously performed using Student's *t*-test, Welch's *t*-test, and χ^2^ tests, where applicable, for the sociodemographic characteristics. The effect size was calculated using Cohen's d, Cramer's V, and Phi, where applicable. The observed differences and associations are reported as statistically significant when *p* < 0.05.

## Results

58.4% of participants (*n* = 342) reported having breastfed their children at the time of the study. Those who did not breastfeed (41.6%, *n* = 244) were asked why 38.9% of them (*n* = 95) stated it was their choice, 36.9% (*n* = 90) did not breastfeed due to lack of breast milk, 25% (*n* = 61) experienced a lactation crisis/stoppage, 9.8% (*n* = 24) were unable to breastfeed due to health issues, 7.8% (*n* = 19) declared other reasons, and 1 participant (0,4%) was convinced not to breastfeed by her family.

## Breastfeeding encouragement

We asked all participants (*N* = 586) if they had ever been encouraged to breastfeed. 81.2% (*n* = 476) answered yes. 18.2% 18 (*n* = 110) declared they had never been encouraged to breastfeed. Those who were incentivized to breastfeed were asked to complete the Breastfeeding Encouragement Questionnaire, in which they rated the sources of encouragement they received. The most substantial encouragement came from healthcare professionals (midwives and doctors), scientific research, and parenting literature, whereas family and friends were less encouraging. The general score of that measure reached 3.44 (SD = 2.32) on a scale from 0 to 9 (see Table 2).

## Pressure to breastfeed

Later, the participants were asked whether they had ever experienced pressure to breastfeed. 39.2% (*n* = 230) reported feeling such pressure, while the majority (60.8%, *n* = 356) had not. Participants who had experienced pressure to breastfeed were asked to complete the Breastfeeding Pressure Questionnaire and rate the potential sources of this pressure. In Table 3, you can see that the most significant pressure comes from mothers themselves, which is exacerbated by doctors and midwives, and by the general idea shared by Polish society that breastfeeding is the best feeding option. The general score of the Breastfeeding Pressure Questionnaire was 3.14 (SD = 2.22) on a scale of 0 to 9. We also asked those participants (*N* = 230) if they still felt pressured during the study. 73.9% (*n* = 170) no longer experienced that feeling, but 26.1% (*n* = 60) still thought they were supposed to breastfeed their children, which shows that most mothers are able to manage that pressure.

To better understand the sources of encouragement and pressure to breastfeed, we conducted a factor analysis of both the Breastfeeding Encouragement Questionnaire and the Breastfeeding Pressure Questionnaire. In both cases, the Kaiser–Meyer–Olkin (KMO) test results showed that the data were well-suited for factor analysis (0.87 and 0.85, *p* < 0.0001, respectively). Again, in both cases, factor analysis using Direct Oblimin rotation with Kaiser normalization extracted two principal factors, which we named the institutional and social components of encouragement and pressure to breastfeed. The analysis showed that the institutional component is dominant (see Table 4). Since the component correlations were substantial (−0.512 and 0.413, respectively), and to keep both Questionnaires easier to use, we decided to continue analyzing both Questionnaires with all items combined.

**Table 4:** Rotated component matrix (rotation converged in seven iterations).

**Source**	**Breastfeeding encouragement questionnaire**		**Breastfeeding pressure questionnaire**	
	**Institutional component**	**Social component**	**Institutional component**	**Social component**
Healthcare professionals	0.708	−0.428	0.674	–
Scientific research results	0.886	−0.450	0.783	0.494
Parenting literature	0.832	−0.437	0.830	–
Breastfeeding promotion campaigns	0.817	–	0.846	0.425
Popular opinion	0.510	0.506	0.758	–
The child's father	0.524	−0.756	0.473	0.734
Parents	0.491	−0.850	–	0.687
Friends	0.499	−0.863	–	0.571
In-laws	–	−0.827	–	0.735
My own expectations	n/a	n/a	0.547	0.503
% of variation explained	51.757%	13.655%	46.177%	12.603%
Component correlation	−0.512		0.413	

We also checked the correlation between the two designed measures. Pearson's r reached 0.78 (*p* < 0.0001), indicating that encouragement to breastfeed is substantially correlated with perceived pressure to breastfeed among Polish mothers of infants.

### Pressure to breastfeed and mothers' self-rating

What is also important is that the Breastfeeding Pressure Questionnaire's overall score showed a weak yet statistically significant correlation with participants' ratings as mothers (*r* = −0.14, *p* = 0.033), suggesting that perceived pressure may negatively impact mothers' mental wellbeing. On the other hand, the average score the mothers rated themselves reached 7.26 (SD = 1.479) on a scale from 0 to 9, which is relatively high. To our surprise, mothers who were breastfeeding gave themselves a lower rating (7.19) than those who were not (7.35), but this difference was not statistically significant (*p* > 0.05). The Breastfeeding Encouragement Questionnaire's general score did not correlate with that variable (*p* > 0.05).

### Pressure to breastfeed and sociodemographic variables

To determine who is more susceptible to the perceived pressure to breastfeed, we analysed the sociodemographic variables. The analysis revealed that women who had experienced the pressure to breastfeed are younger than those who had not (see Table 5).

**Table 5:** Age and the perceived pressure to breastfeed (***N*** = 586).

**Experienced pressure to breastfeed**		**Mean age**	**SD**
Experienced pressure to breastfeed	Yes	28.98	4.28
	No	31.03	4.63

*t*_(584)_ = −5.371, *p* < 0.0001, Cohen's d = −0.45.

Table 6 presents the data on the frequency of feeling pressured to breastfeed in comparison with other sociodemographic variables. The results indicate that women residing in rural environments experience pressure more frequently than those residing in towns and cities. The level of education did not differentiate the frequency of experiencing pressure to breastfeed [$\chi_{\left(2\right)}^{2}$ = 2.80, *p* > 0.05]. However, the study revealed that women who had their first baby tended to feel more pressure than those who had their second or third baby. But what seems to be most important is that women who did not breastfeed due to various reasons tended to feel the pressure more often than those who did breastfeed. More than half (57.4%) of the women who were not breastfeeding their infants at the time of the study reported experiencing pressure. At the same time, only 26.3% of breastfeeding mothers reported the same experience.

**Table 6:** Sociodemographic variables and the experienced pressure to breastfeed (*N* = 586).

**Questionnare factors**		**Experienced pressure to breastfeed**		**Statistical tests**
		**Yes**	**No**	
Place of residence	Rural areas	47.1%	52.9%	χ^2^ = 11,323, *p* = 0.023, Cramer's *V* = 0.139
	A town with up to 50 thousand inhabitants	33.0%	67.0%	
	A city with between 50 and 150 thousand inhabitants	41.4%	58.6%	
	A city with between 150 and 500 thousand inhabitants	42.5%	57.5%	
	A city with >500 thousand inhabitants	31.9%	68.1%	
First child	Yes	42.6%	57.4%	χ^2^ = 5.374, *p* = 0.020, phi = 0.09
	No	32.7%	67.3%	
Currently breastfeeding	Yes	26.3%	73.7%	χ^2^ = 57.62, *p* = 0.0001, phi = −0.31
	No	57.4%	42.6%	

However, what seems very interesting is that the sociodemographic variables do not differentiate the values of the Breastfeeding Pressure Questionnaire general score (*p* > 0.05).

The research team wanted to check if mothers who did not breastfeed experienced the pressure differently from breastfeeding mothers. Since there was no difference between those two groups in the Breastfeeding Pressure Questionnaire (Welch's *t*_(210)_ = 0.39, *p* > 0.05), we decided to investigate each item of the Questionnaire separately. This analysis showed that among participants who had experienced pressure to breastfeed (*N* = 230), mothers who were not breastfeeding their children at the time of the study perceived the sources of that pressure differently than those who were. They were putting less pressure on themselves and experiencing less pressure from their children's fathers, but at the same time, they felt greater pressure from doctors and midwives (see Table 7).

**Table 7:** Pressure to breastfeed sources rating (scale from 0 to 9) on breastfeeding and non-breastfeeding mothers (*N* = 230).

**Currently breastfeeding**	Healthcare professionals^*^	Own expectations^**^	The child's father^***^
Yes	4.14	6.24	2.63
No	5.11	4.14	1.45

^*^t_(228)_ = −2.006, *p* = 0.046, Cohen's d = −0.27; ^**^t_(228)_ = 4.365, *p* < 0.001, Cohen's d =0.59; Welch's *t*_(157)_ = 2.900, *p* < 0.005, Cohen's d = 0.401.

## Discussion

The presented results suggest that Poland is an example of a ‘breastfeeding culture' (14). At some point, over 80% of the participants were encouraged to breastfeed their children, mainly by healthcare professionals but also by the information the mothers could find themselves (scientific research, parenting literature, breastfeeding promotion campaigns) and the popular opinion that ‘breast is best,' which may imply that formula-feeding is worse. Women may start to believe that if they are not successfully and exclusively breastfeeding, they may not be doing what is best for their children (20), which may lead to experiencing public stigma (21), failure (22), guilt (23), and pressure to breastfeed (24). Our study reveals that nearly 40% of mothers of infants who participated in the survey reported experiencing this pressure, and 10.24% (*n* = 60) felt it during their participation in the study. Those numbers may seem low compared to the results of a similar study conducted in the USA, where the number of mothers reporting pressure to breastfeed reached 82% (25). One reason the frequency levels differ may be that the American study focused on new mothers, while our study included mothers with children before, who tend to experience less pressure to breastfeed. However, both studies show that the most substantial pressure to breastfeed comes from mothers' own expectations. This proves that the ‘breastfeeding culture' is deeply internalized by women both in Poland and the USA. In the Polish case, this may be attributed to the long-standing promotion of breastfeeding, which dates back to the 1990s (26).

The second most potent source of pressure, which was recognized as the primary source of encouragement to breastfeed, is the doctors and midwives. It may be so since, according to other studies, healthcare personnel present breastfeeding as the best feeding method and focus mainly on achieving it, regardless of the needs and feelings of the mother, without presenting the pros and cons of other feeding options, which sometimes results in a sense of pressure to breastfeed (18, 27, 28).

According to our results, several characteristics make women more susceptible to experiencing that pressure. Younger mothers, those residing in rural areas, and those raising their first-born children reported the pressure more frequently. However, the experience of pressure to breastfeed was most frequent among non-breastfeeding mothers. Our study also showed that experiencing the pressure to breastfeed correlates with lower ratings the participants gave themselves as mothers. These findings are consistent with the results of prior research, which show that problems with breastfeeding can lead to feelings of pressure and negatively impact maternal mental health by inducing increased anxiety, stress, and birth trauma (8, 17, 25, 29, 30).

Our findings can also be interpreted through the lens of the Theory of Planned Behavior (TPB) which states that intention, which is a predictor of actual behavior, is shaped by attitudes towards the behavior (positive or negative), subjective norms (the perceived social pressure to perform or not to perform the behavior), and perceived behavioral control (the perceived ease or difficulty of performing the behavior) (31). The strong influence of mothers' own expectations and healthcare professionals as sources of pressure reflects the role of subjective norms—both internalized (personal standards) and external (social and professional expectations)—in shaping breastfeeding intentions. Similar patterns have been observed in prior TPB-based studies, where normative beliefs from family and health professionals have been found to significantly predict breastfeeding initiation and continuation (32, 33). The high correlation between encouragement and perceived pressure (*r* = 0.78) suggests that normative beliefs in Poland favor breastfeeding, creating an environment where deviation from this norm may evoke guilt or lower one.'.

## Limitations of the study

The study's main limitations include its cross-sectional design, the use of a brief and invalidated research tool, and the online sampling method. Another issue is that the results of the designed measures, the Breastfeeding Encouragement Questionnaire and the Breastfeeding Pressure Questionnaire, have not been correlated with existing scales measuring breastfeeding experience. The reason for this was the need to keep the designed survey as brief as possible, as people in Poland are becoming increasingly reluctant to participate in surveys, and a long questionnaire would discourage them even more. Unfortunately, the research team did not have the resources to provide any incentives for the participants; therefore, we decided to limit the number of questions (34, 35).

However, despite a considerably large sample size, due to the abovementioned limitations, the study's results should be interpreted as exploratory. Nevertheless, to the best of our knowledge, this research is the first attempt to investigate perceived pressure to breastfeed among mothers in Poland and should be viewed as an exploratory study that lays the groundwork for future, more advanced research that will help to understand the phenomenon of pressure to breastfeed better and to refine both designed research tools.

## Conclusions

The feeling of pressure to breastfeed is quite common among mothers of infants in Poland; however, its level is not particularly high. The pressure makes the ones who experience it feel like lesser mothers and can have a negative impact on their mental health. Doctors and midwives were identified as one of the primary sources of that pressure; therefore, it is important that healthcare professionals pay more attention to presenting breastfeeding in a more nuanced way, especially to younger, inexperienced mothers. They should also support those mothers who experience problems with breastfeeding or cannot breastfeed at all, and do as much as possible to take the pressure off of them.

## Data Availability

The raw data supporting the conclusions of this article will be made available by the authors, without undue reservation.
